# The Israeli National Genetic database: a 10-year experience

**DOI:** 10.1186/s40246-017-0100-z

**Published:** 2017-03-16

**Authors:** Joël Zlotogora, George P. Patrinos

**Affiliations:** 1 Faculty of Medicine, Hebrew University, Jerusalem, Israel; 2 Department of Pharmacy, University of Patras School of Health Sciences, University Campus, Rion, 26504 Patras, Greece; 3 Department of Bioinformatics, School of Medicine and Health Sciences, Erasmus University Medical Center Rotterdam, Rotterdam, The Netherlands

**Keywords:** Israel, Arabs, Database, Genetic disorders, Founder effect

## Abstract

**Background:**

The Israeli National and Ethnic Mutation database (http://server.goldenhelix.org/israeli) was launched in September 2006 on the *ETHNOS* software to include clinically relevant genomic variants reported among Jewish and Arab Israeli patients. In 2016, the database was reviewed and corrected according to ClinVar (https://www.ncbi.nlm.nih.gov/clinvar) and ExAC (http://exac.broadinstitute.org) database entries. The present article summarizes some key aspects from the development and continuous update of the database over a 10-year period, which could serve as a paradigm of successful database curation for other similar resources.

**Results:**

In September 2016, there were 2444 entries in the database, 890 among Jews, 1376 among Israeli Arabs, and 178 entries among Palestinian Arabs, corresponding to an ~4× data content increase compared to when originally launched. While the Israeli Arab population is much smaller than the Jewish population, the number of pathogenic variants causing recessive disorders reported in the database is higher among Arabs (934) than among Jews (648). Nevertheless, the number of pathogenic variants classified as founder mutations in the database is smaller among Arabs (175) than among Jews (192). In 2016, the entire database content was compared to that of other databases such as ClinVar and ExAC. We show that a significant difference in the percentage of pathogenic variants from the Israeli genetic database that were present in ExAC was observed between the Jewish population (31.8%) and the Israeli Arab population (20.6%).

**Conclusions:**

The Israeli genetic database was launched in 2006 on the *ETHNOS* software and is available online ever since. It allows querying the database according to the disorder and the ethnicity; however, many other features are not available, in particular the possibility to search according to the name of the gene. In addition, due to the technical limitations of the previous ETHNOS software, new features and data are not included in the present online version of the database and upgrade is currently ongoing.

## Background

The Israeli population includes Jewish and Arab communities, in each of which genetic disorders have been reported in a relatively high frequency [1]. Among Jews, the late Professor Richard Goodman compiled relatively frequent genetic disorders and published a seminal book “Genetic disorders among the Jewish people” [2]. On the basis of this book, a catalog of genetic disorders in the Jewish population was created in 1998 and later, a catalog of genetic disorders in the non-Jewish population in Israel was added. The data included in these two catalogs were updated at least once a year. In 2006, this compilation gave rise to the Israeli National Genetic database [3], a freely available online resource for genetic services in Israel. This database has resulted from a customized version of the *ETHNOS* software, adapted to accommodate large datasets and to support both menu- and keyword-based queries. Ever since, the Israeli national and ethnic mutation database (NEMDB) has become a useful online resource for genetic services in Israel.

In 2016, the Israeli population included 8,556,000 citizens of whom 74.8% were Jews and 20.8% Arabs [4]. Among Israeli Arabs, 83.1% were Muslim, 9.7% Christian Arabs, and 7.8% Druze. The Bedouins that have been a nomad population represent one fifth of the Muslim Arabs and in a majority live in the Negev desert. Jewish communities emigrated from many countries in the world joining the Jewish population living in Israel. While differences existed between the various regions in Europe where the Ashkenazi Jews were living, it is difficult to distinguish subgroups among them. On the other hand, most of the other Jewish communities remained distinct.

## Implementation

The Israeli National Genetic database (http://server.goldenhelix.org/israeli) includes causative genomic variants that were characterized among affected patients either Israeli Arabs or Jews living in Israel and in the diaspora. Palestinian Arabs living under the Palestinian Authority, or for whom details on the exact origin were not available, were recently added as a distinct population. All recessive mutations are included while dominant or X-linked mutations are recorded in the database either if they are founder mutations, or if they were reported in several families, or if the disorder is relatively frequent. Since the launch of the database, each entry consists of the name of the disorder (usually as used in the Online Mendelian Inheritance in Man (OMIM); [https://www.ncbi.nlm.nih.gov/omim]), the name of the gene and its OMIM number, and the name of the mutation, whether it was reported as a single allele, a family, several families or is a founder mutation. In addition, the entry includes details on the origin of the patient if Jewish either Ashkenazi or the country of origin and if Arab the religion either Druze Christian or Muslim and the locality of origin. The Muslim Arabs known to be Bedouins are entered as a separate subgroup. The frequency of the mutation in the specific population is recorded when available.

The information included in the database is updated according to publications in the scientific literature and personal communications. When details needed for the database are not included in the original report, the authors are contacted in order to complete the data if available. Since the end of 2010, the source of the data was added to each new entry and when possible, this was done also for older data.

In 2016, the entire database was reviewed and corrected according to ClinVar (https://www.ncbi.nlm.nih.gov/clinvar) and ExAC (http://exac.broadinstitute.org) database entries. Data from ClinVar were added to the database for each mutation including, when available, genomic location, rs number, pathogenicity, and OMIM reference for the mutation. The frequency in ExAC as calculated on 60,706 unrelated individuals was added for the variants documented in ExAC [5].

## Results

### Database entries

In September 2016, there were 2444 entries in the database, 890 among Jews, 1376 among Israeli Arabs, and 178 entries among Palestinian Arabs. The data on Palestinian Arabs are scarce since added only recently and therefore were not included in the present analysis. Among the 890 entries in Jews, there were 783 that were different mutations; the other entries were cases of a same pathogenic variant present in more than one community. There were 357 entries among the Ashkenazi Jews, 86 entries among Moroccan Jews, 71 entries among Iraqi Jews, 57 entries among Iranian Jews, 47 entries among Yemenite Jews, and 260 entries among specific smaller communities. In addition, there were 98 entries in three Jewish subgroups for which details were not available: non-Ashkenazi (38 entries), Sephardi (11 entries), or North African (21 entries) and a group of Jews of unknown origin (28 entries). These data are summarized in Table 1.

**Table 1:** Data content statistics from the entire Israeli NEMDB

	Entries	Unique entries	ExAC (% unique entries)	ExAC >0.5 per 1000
Jews	890	783	247 (31.8%)	44 (5.8%)
Ashkenazi	357	357	136 (38.9%)	28 (7.8%)
Morocco	86	86	34 (39.5%)	8 (9.3%)
Iraq	71	71	20 (28.2%)	8 (11.7%)
Iran	57	57	19 (33.3%)	2 (2.8%)
Yemen	46	46	8 (17.0%)	1 (2.1%)
Israeli Arabs	1376	977	201 (20.6%)	22 (2.2%)
Christian	102	102	19 (18.6%)	4 (3.9%)
Muslim	814	814	151 (18.6%)	19 (2.3%)
Bedouins	253	253	36 (14.2%)	4 (1.6%)
Druze	108	108	19 (17.6%)	1 (1.9%)

The total numbers of entries among Jews include similar entries reported in more than one community, and entries in small communities are not included in the Table. Among Arabs, the total number of entries includes duplicated entries in which a same pathogenic variant was reported in more than one locality in patients from a same religious group and similar entries reported in more than one community

Among the Israeli Arabs, there were 1376 entries, 99 being duplicated since a same pathogenic variant was reported in more than one locality in patients from a same religious group. There were 102 unique entries among Christian Arabs and 108 among Druze. Among the Muslim Arabs, there were 814 unique entries with an additional 253 entries in Muslims known to be of Bedouin origin. Among the 1277 non-duplicated entries, the village of origin was known in 1075 cases.

### Frequency of the pathogenic variants as reported in ExAC

Among the 783 unique entries in Jews, the pathogenic variant was present in ExAC in 247 entries (31.8%) and the frequency of the pathogenic variant was 0.5% or more for 44 of them (5.8%). Among the 977 unique entries in Israeli Arabs, the pathogenic variant was present in ExAC in 201 cases (20.6%) and the frequency of the pathogenic variant was 0.5% or more for 22 of them (2.2%).

Details on the presence of the pathogenic variant and their frequency in ExAC in the different Jewish and Israeli Arab communities are given in Table 1.

### Autosomal recessive disorders in the Israeli NEMDB

Subsequently, we sought to analyze the number of pathogenic variants that were reported among patients affected with autosomal recessive disorders among Jews and Israeli Arabs. Among the Jews, there were 648 different pathogenic variants in a total of 306 different genes, from which 192 being founder mutations (29.6%). In 135 out of the 306 genes, more than one pathogenic variant was reported (44.1%), and in 77 out of these genes, at least one was a founder mutation. Among the Israeli Arabs, there were 934 different pathogenic variants in a total of 473 different genes, 175 being founder mutations (18.7%). In 151 out of the 473 genes, more than one pathogenic variant was reported (31.9%), and in 61 out of these genes, at least one was a founder mutation. The same parameters were looked for in the different Jewish and Israeli Arab communities, and the data are summarized in Table 2.

**Table 2:** Autosomal recessive disorders reported in affected patients

	Unique entries	Number of genes	Genes with >1 pathogenic variant^a^	Founder mutations^b^	Founder in >1 mutation^c^
JEWS	648	306	135 (44.1%)	192 (29.6%)	77 (57%)
Ashkenazi	283	161	61 (37.9%)	66 (23.3%)	44 (72.1%)
Morocco	80	48	17 (35.4%)	32 (40%)	13 (76.5%)
Iraq	50	44	11 (22.9%)	23 (46%)	8 (72.7%)
Iran	62	43	3 (7%)	23 (37.1%)	3 (100%)
Yemen	37	31	4 (13.3%)	7 (18.9%)	2 (50%)
Israeli Arabs	934	473	151 (31.9%)	175 (18.7%)	61 (40.4%)
Christian	58	38	7 (17.5%)	6 (10.3%)	3 (48.3%)
Muslim	553	335	89 (26.6%)	89 (16.1%)	33 (37.1%)
Bedouins	170	140	25 (17.9%)	86 (50.6%)	20 (80%)
Druze	75	58	12(20.7%)	19 (25.3%)	7 (58.3%)

The total numbers of entries among Jews include similar entries reported in more than one community, and entries in small communities are not included in the Table. Among Arabs, the total number of entries includes duplicated entries in which a same pathogenic variant was reported in more than one locality in patients from a same religious group and similar entries reported in more than one community

^a^The percentage in parenthesis is the number of genes with more than one pathogenic variant out of the number of genes

^b^The percentage in parenthesis is the number of founder mutations out of the number of unique entries

^c^The percentage in parenthesis is the number of genes with more than one founder mutation out of the number of genes with more than one pathogenic variant

### Pathogenic variants present in more than one community

We then looked for pathogenic variants that were present in more than one community. In 52 instances, the same pathogenic variant was found among Jews and Arabs out of which 37 variants were present in the ExAC database (71.2%). Twenty-five out of these 37 variants (67.6%) were found in Ashkenazi Jews, from which 23 variants were reported in the ExAC database (92%).

In 17 instances, the same pathogenic variant was found in geographically close Jewish communities, including 6 among North African communities and 11 among Eastern Jewish communities. In 32 cases, the same pathogenic variant was found in geographically distant Jewish communities, 17 of which were documented in ExAC (53.1%). In 19 of these 32 cases, the pathogenic variant was found in Ashkenazi Jews, 11 of which were also documented in ExAC (57.9%).

In 16 cases, a pathogenic variant was reported in more than one Arab religious community but not among Jews. Among these 16 cases, 6 pathogenic variants were reported in ExAC (37.5%).

## Discussion

The technology revolution in genomic analysis changed the ability for diagnosis and characterization of genetic diseases in the last decades. While the late Richard M Goodman in 1979 delineated only 11 monogenic disorders relatively frequent in the Ashkenazi Jews [2], in September 2016, founder mutations were reported in the Ashkenazim responsible for 62 autosomal recessive disorders, 7 dominant disorders, and one X-linked disease. Since the creation of the Israeli NEMDB in September 2006, the number of database records has been almost quadrupled. For instance, new founder mutations were added in 36 genes among the Ashkenazi Jews in 10 years since the launch of the Israeli NEMDB.

The Ashkenazi represents the largest Jewish community in Israel and in the diaspora, and therefore, the observation that most of the entries among Jews (63%) are in this community was not unexpected. However, while the Israeli Arab population is much smaller than the Jewish population, the number of pathogenic variants causing recessive disorders reported in the database is higher among Arabs (934) than among Jews (648). Nevertheless, the number of pathogenic variants classified as founder mutations in the database is smaller among Arabs (175) than among Jews (192), which can be explained by pathogenic variant expansion in isolated populations.

Whole genome sequencing of random individuals and their parents have demonstrated that every individual is born with 44–82 de novo single-nucleotide mutations [6], and therefore, in a defined population, many of the newborns are carriers of new variants responsible for recessive diseases. In a previous study, the fate of recessive mutations was followed in an Israeli Muslim village in which the families are large and close consanguinity is frequent [7]. In this village, a new variant, occurring de novo or being introduced by marriage of a carrier from another village, may appear in homozygosity in a patient already after three generations. In such isolated populations, some of the new variants spread within the kindred of the first carrier, either randomly or as the result of a selective advantage, and later may become founder mutations within the community. A change in the marriage patterns such as marriages outside of the isolate and smaller size of the families will reduce the number of patients affected due to founder mutations. In parallel, some of the founder mutations that were present only in isolated communities will appear at a lower frequency in the whole population. Before the creation of the state of Israel, the Jewish communities were isolated one from the other because of geographical distances and from the surrounding populations by the preference of marriages within the religious community and often within the family. As a result, among Jews, founder mutations are found in the database in each of the different communities. Indeed, few of these founder Jewish mutations are present in more than one community, mainly since they occurred before the dispersion of the Jews. For instance, the mutation p.F301L in F11 which is nowadays frequent among Ashkenazi Jews and Jews from Iraq was probably present among Jews already 2.5 millennia ago [8]. Among the Israeli Arabs, the preference of marriages within the close family and within the religious community that was responsible for their isolation is still predominant nowadays. Therefore, among the Israeli Arabs, founder mutations are still mostly limited to single villages or tribes. There are three exceptions in the database: one is the p.T322X mutation in the *ERCC8* gene causing Cockayne syndrome that was shown to be frequent in the entire Christian Arab community and has been reported in Christian Arabs from Lebanon [9]. The second exception is the p.S52_G55del mutation in the *TBCE* gene causing hypoparathyroidism and mental retardation that originated among Bedouins in Saudi Arabia who are at the origin of the Israeli Bedouin Arabs [10]. The third exception is a mutation present in all the Israeli Arab Bedouins p.P615Sfs*12 in the *NTRK1* gene causing congenital insensitivity to pain with anhidrosis but has not been reported in other populations and therefore probably occurred more recently [11]. In Israel, many changes are occurring both in the Jewish and Arab populations in particular of the marriage patterns that while they remain within the religious community are more often outside the isolate. This is particularly frequent among Jews since consanguinity became rare since the creation of the State of Israel and intercommunity marriages became frequent [12]. Among Arabs, the changes are slower and consanguinity is still preferred, but marriages outside the village became more and more frequent [13]. In both cases, these changes are expected to scatter the founder mutations. The ultimate result that may be expected is that some founder mutations will remain and will become Israeli Jewish or Israeli Arab mutations existing in each population at lower frequency.

A significant difference in the percentage of pathogenic variants from the Israeli NEMDB that were present in ExAC was observed between the Jewish population (31.8%) and the Israeli Arab population (20.6%). The difference between the Ashkenazi Jews and the Israeli Arabs was expected since the European population including Ashkenazi Jews is better represented in ExAC than the Middle East population [5]. However, the Jews from Morocco, Iran, and Iraq have a percentage of mutations present in ExAC in the same range as the Ashkenazi Jews even though these communities originated from populations that are not well represented in ExAC. This may in part be explained by the observation that several variants found among Ashkenazi Jews and present in ExAC were also characterized in other Jewish communities. For instance, among the 34 variants found in Moroccan Jews that were present in ExAC, 8 were variants that are also found among Ashkenazi Jews. Similarly, among the mutations common to Jews and Israeli Arabs, those reported among Ashkenazi Jews were almost always present in ExAC (92%).

## Conclusion

The Israeli NEMDB was launched in 2006 on the ETHNOS software and is available online ever since. It allows querying the database according to the disorder and the ethnicity; however, many other features are not available, in particular the possibility to search according to the name of the gene. In addition, due to the technical limitations of the previous ETHNOS software on which the Israeli NEMDB runs, new features and data are not included on the present online version of the database such as the source of the data, genomic location of the variant, it rs number, and OMIM reference or the effect of the mutation as described in the ClinVar database. Upgrade of the Israeli NEMDB is currently ongoing in order to include all the data described in the article and allow new querying possibilities.

## Abbreviations

NEMDB: National and Ethnic Mutation Database
OMIM: Online Mendelian Inheritance in Man
